# Human mesenchymal stem cells - current trends and future prospective

**DOI:** 10.1042/BSR20150025

**Published:** 2015-04-28

**Authors:** Imran Ullah, Raghavendra Baregundi Subbarao, Gyu Jin Rho

**Affiliations:** *Department of Theriogenology and Biotechnology, College of Veterinary Medicine, Gyeongsang National University, Jinju 660-701, Republic of Korea; †Research institute of life sciences, Gyeongsang National University, Jinju 660-701, Republic of Korea

**Keywords:** chronic diseases, homing, immunomodulatory features, *in vitro* differentiation, mesenchymal stem cells, AD, Alzheimer disease, AD-MSC, adipose-derived mesenchymal stem cell, ALS, amylotrophic lateral sclerosis, BDNF, brain-derived neurotrophic factor, BME, β-mercaptoethanol, BM-MSC, bone marrow-derived mesenchymal stem cell, BMP, bone morphogenic protein, CD, cluster of differentiation, CPA, cryoprotective agent, CRF, controlled rate freezer, DA, dopamine, DMEM, Dulbecco's modified Eagle's media, EGF, epidermal growth factor, ESC, embryonic stem cell, FCS, fetal calf serum, FGF, fibroblast growth factor, HGF, hepatocyte growth factor, HLA, human leucocyte antigen, hMSC, human mesenchymal stem cell, ICM, inner cell mass, IFN, interferon, IL, interleukin, IMDM, Iscove's modified Dulbecco's medium, iPSC, induced pluripotent stem cell, LMX1a, LIM homoeobox transcription factor 1 α, MHC, major histocompatibility complex, MMP, matrix metallo-protease, MSC, mesenchymal stem cell, NBCS, new-born calf serum, NK, natural killer, PD, Parkinson's disease, PD, population doubling, PPARγ, peroxisome proliferator-activated receptor γ, RA, rheumatoid arthritis, Runx2, runt-related transcription factor 2, SSEA, stage-specific embryonic antigen, TGF-β, transforming growth factor-β, Th, T helper cell, TLR, toll-like receptor, Treg, regulatory T-cell, UCB-MSC, umbilical cord blood-derived mesenchymal stem cell

## Abstract

Stem cells are cells specialized cell, capable of renewing themselves through cell division and can differentiate into multi-lineage cells. These cells are categorized as embryonic stem cells (ESCs), induced pluripotent stem cells (iPSCs) and adult stem cells. Mesenchymal stem cells (MSCs) are adult stem cells which can be isolated from human and animal sources. Human MSCs (hMSCs) are the non-haematopoietic, multipotent stem cells with the capacity to differentiate into mesodermal lineage such as osteocytes, adipocytes and chondrocytes as well ectodermal (neurocytes) and endodermal lineages (hepatocytes). MSCs express cell surface markers like cluster of differentiation (CD)29, CD44, CD73, CD90, CD105 and lack the expression of CD14, CD34, CD45 and HLA (human leucocyte antigen)-DR. hMSCs for the first time were reported in the bone marrow and till now they have been isolated from various tissues, including adipose tissue, amniotic fluid, endometrium, dental tissues, umbilical cord and Wharton's jelly which harbours potential MSCs. hMSCs have been cultured long-term in specific media without any severe abnormalities. Furthermore, MSCs have immunomodulatory features, secrete cytokines and immune-receptors which regulate the microenvironment in the host tissue. Multilineage potential, immunomodulation and secretion of anti-inflammatory molecules makes MSCs an effective tool in the treatment of chronic diseases. In the present review, we have highlighted recent research findings in the area of hMSCs sources, expression of cell surface markers, long-term *in vitro* culturing, *in vitro* differentiation potential, immunomodulatory features, its homing capacity, banking and cryopreservation, its application in the treatment of chronic diseases and its use in clinical trials.

## Abstract 2

In this review, we highlighted recent research findings in the area of human mesenchymal stem cells, its application in the treatment of chronic diseases and its use in human clinical trials.

## INTRODUCTION

Stem cells are the cells with a specific function with the ability of self-renewal, possess varied potency and differentiate into multilineages [1]. Because of clinical applications and biological importance, stem cells become a prominent subject in modern research era. On the basis of origin, stem cells are divided into different categories.

Embryonic stem cells (ESCs) are pluripotent stem cells, isolated originally from the inner cell mass (ICM) of mouse early pre-implantation blastocyst, having the capacity to generate into any mature cell of the three germ lines [2]. Later on, Thomson et al. [3] also isolated ESCs from ICM of human blastocyst, but until now as compared with humans, only mouse ESCs have been investigated in depth. ESCs possess distinctive self-renewal capacity, pluripotency and genomic stability [4] and can give rise to almost all lineages and are promising cells for cellular therapy [1]. From the very first derivation of human ESCs, scientists are keenly interested in the use of ESCs for drug discovery, immunotherapy and regenerative medicine, but their use has been restricted due to ethical issues and also because of difficulty in obtaining quality human oocytes.

Induced pluripotent stem cells (iPSCs) are generated from adult cells by the overexpression of four transcription factors Oct4/3 (octamer-binding transcription factor 4/3), Sox2 (sex determining region Y), Klf4 (kruppel-like factor 4) and c-Myc (Avian Myelocytomatosis virus oncogene cellular homologue) [5]. The iPSCs at cellular level are almost similar to ESCs as they are having the capacity of self-renewal, differentiation potential and the ability to produce germ line competent-chimeras. After these findings, two groups Takahashi et al. [6] and Nakagawa et al. [7] have generated the iPSCs from adult human fibroblasts. Though iPSCs possess great potential for cell therapy, but their genomic stability is still questionable.

Around the world, scientists are researching for stable, safe and highly accessible stem cells source with great potential for regenerative medicine. The cells isolated from mouse bone marrow upon culture exhibited the plastic adherence properties and formed spindle-shaped colonies were referred as colony forming unit fibroblasts [8]. Due to their ability to differentiate into specialized cells developing from mesoderm, they were named as mesenchymal stem cells (MSCs). MSCs, also known as multipotent cells, exist in adult tissues of different sources, ranging from murine to humans. They are self-renewable, multipotent, easily accessible and culturally expandable *in vitro* with exceptional genomic stability and few ethical issues, marking its importance in cell therapy, regenerative medicine and tissue repairment [9].

The current review highlights recent findings in the areas of hMSCs (human MSCs) sources, its *ex vivo* differentiation ability, immunogenicity, homing ability, banking and cryopreservation, its role in the treatment of chronic diseases and its use in human clinical trials.

## HUMAN MESENCYMAL STEM CELLS

Since the first description of hMSCs derived from bone marrow [10], they have been isolated from almost all tissues including perivascular area [11]. Still there is neither a single definition nor a quantitative assay to help in the identification of MSCs in mixed population of cells [9]. However, the International Society for Cellular Therapy has proposed minimum criteria to define MSCs. These cells (a) should exhibits plastic adherence (b) possess specific set of cell surface markers, i.e. cluster of differentiation (CD)73, D90, CD105 and lack expression of CD14, CD34, CD45 and human leucocyte antigen-DR (HLA-DR) and (c) have the ability to differentiate *in vitro* into adipocyte, chondrocyte and osteoblast [12]. These characteristics are valid for all MSCs, although few differences exist in MSCs isolated from various tissue origins.

### Sources

MSCs are present not only in fetal tissues but also in many adult tissues with few exceptions. Efficient population of MSCs has been reported from bone marrow [10]. Cells which exhibits characteristics of MSCs were isolated from adipose tissue [13,14], amniotic fluid [15,16], amniotic membrane [17], dental tissues [18,19], endometrium [20], limb bud [21], menstrual blood [22], peripheral blood [23], placenta and fetal membrane [24], salivary gland [25], skin and foreskin [26,27], sub-amniotic umbilical cord lining membrane [28], synovial fluid [29] and Wharton's jelly [30,31] (Table 1).

**Table 1:** Summary of hMSCs sources, cell surface markers and expansion media with serum supplements

Source	Method of isolation	Media	Serum supplement	Cell surface markers		References
Bone marrow	Ficoll density gradient method Novel marrow filter device	DMEM DMEM-F12 ADMEM	FBS	Positive	Negative	[10,36,38,47,48]
				CD73, CD90, CD105, STRO-1	CD14, CD34, CD45, HLA-DR	
Adipose tissue	Digestion method Membrane filtration method	DMEM DMEM-LG	FBS FCS	CD73, CD090, CD29, CD44, CD71, CD105, CD13, CD166, STRO-1	CD14, CD31, CD34, CD45	[13,34,43,49,57]
Amniotic fluid and membrane	Density gradient method Digestion method	α-MEM DMEM/F12	FBS	CD29, CD44, CD90, CD105, CD, SH2, SH3, HLA-DR	CD10, CD14,CD34, HLA-DR	[15–17]
Dental tissues	Digestion method	α-MEM MEM	FCS FBS	CD29, CD44, CD90, CD105	CD14, CD34, CD45	[18,19,46]
Endometrium	Digestion method	DMEM-F12	FCS	CD73, CD90, CD105, CD146	CD34, CD45	[20]
Limb bud	Digestion method	DMEM-LG	FBS	CD13, CD29,CD90, CD105, CD106	CD3, CD4, CD14, CD15, CD34, CD45, HLA-DR	[21]
Peripheral blood	Ficoll density gradient	α-MEM	NBCS	CD44, CD90, CD105, HLA-ABC	CD45, CD133	[23]
Placenta and fetal membrane	Digestion method	DMEM-LG	FBS	CD29, CD73, CD90, CD105	CD34, CD45	[24]
Salivary gland	Digestion method (Ringer solution)	DMEM	FCS	CD13, CD29, CD44, CD90, STRO-1	CD34, CD45	[25]
Skin and foreskin	Digestion method	DMEM-HG DMEM DMEM-F12	FBS	CD44, CD73, CD90, CD105, CD166, SSEA-4, Vimentin	CD34, CD45, HLA-DR	[26,27]
Sub amniotic umbilical cord lining membrane	Digestion method	DMEM-HG DMEM CMRL1660	FBS	CD29, CD44, CD73, CD90, CD105	CD34, CD45	[13,28,58]
Synovial fluid	Ficoll density gradient method	α-MEM	FBS	CD44, CD90, CD105, CD147, STRO-1	CD31, CD34, CD45, CD106	[29]
Wharton's jelly	Enzymatic digestion method	DMEM	FBS	CD73, CD90, CD105	CD14, CD34, CD45, CD79, HLA-DR	[31,32]

### Isolation and initial culturing

There are different protocols reported previously in terms of isolation, characterization and expansion of MSCs, but all MSCs (despite of protocol) exhibits the minimum criteria proposed by International Society for Cellular Therapy.

hMSCs were isolated based on their ability to adhere to plastic surface, but this method resulted in the formation of heterogeneous cells (stem cells along with their progenitor cells) [32]. Bone marrow-derived MSCs (BM-MSCs) are considered the best cell source and taken as a standard for the comparison of MSCs from other sources.

Establishment of a comprehensive procedure for the isolation, characterization and expansion of MSCs is the key to success for the use of these cells as a good source for regenerative medicine [33]. Unlike bone marrow, MSCs from other tissues can be easily obtained by non-invasive methods and its proliferation can be maintained up to many passages [34,35]. MSCs from bone marrow, peripheral blood and synovial fluid were isolated by using Ficoll density gradient method with small modifications [24,30,36] and seeded into culture plates. While isolating MSCs from bone marrow, some haematopoietic cells also adhere to the plastic plate but during sub-culturing these cells are washed away, leaving only adherent fibroblast like cells [37]. MSCs from various tissue sources (adipose, dental, endometrium, foreskin, placenta, Wharton's Jelly) were isolated after digestion with collagenase and then cultured at varying densities [20,25,33]. Recently an efficient method to isolate BM-MSCs using novel marrow filter device is explored [38], which is less time consuming and avoids the risk of external contamination. MSCs isolated from different sources were cultured using condition media such as Dulbecco's modified Eagle's media (DMEM) [25,33], DMEM-F12 [17,20,26], αMEM [19,23,29], DMEM-LG [21,24], DMED-HG [27,28] and RPMI (Roswell Park Memorial Institute medium) [39]. The primary culture media was supplemented with 10% FBS [25,33], new-born calf serum (NBCS) [23] or fetal calf serum (FCS) [25] (Table 1). Besides the culture media and supplementation, the oxygen concentration also affects the expansion and proliferation of MSCs [40]. MSCs expansion is also documented when cultured in DMEM with low glucose supplemented with growth factors like fibroblast growth factor (FGF), epidermal growth factor (EGF) and B27 [27]. But most commonly DMEM with 10% FBS is vastly employed in culturing and expanding MSCs *in vitro*; however, the use of exogenous FBS is highly debated.

### Expression of cell surface markers

According to the International Society for Cellular Therapy standard criteria, expression of specific set of cell surface markers is one of the essential characteristics of hMSCs. Those cells which are positive for CD73, D90, CD105 whereas negative expression of CD14, CD34, CD45 and HLA-DR are considered as MSCs. However, the most characterized and promising markers with highest specificities for MSCs are describe in the present study (Table 1). MSCs have been reported from various human tissues, which exhibit the expression of above mentioned cell surface markers along with positive expression of CD29, CD44, CD146, CD140b specific to tissue origin. The expression of CD34, which is a negative marker, is still controversial [41]. A number of studies have also reported that stage-specific embryonic antigen (SSEA)-4 [13,42], CD146 [43,44] and stromal precursor antigen-1 (Stro-1) [45] are the stemnes markers for MSCs. The human amniotic fluid-derived MSCs exhibits the expression of CD29, CD44, CD90, CD105, HLA-ABC [major histocompatibility complex class I (MHC I)] along with SH2 (Src homology 2), SH3 (Src homology 3), SH4 (Src homology 4) but lack the expression of HLA-DR (MHC II) [16]. Stro-1, which is consider as stemnes marker for MSCs, is reported positive in dental [46] and bone marrow [47,48] whereas negative in human adipose-derived MSCs (AD-MSCs) [49].

### Long-term *in vitro* culturing capacity

Although MSCs have great advantages over other stem cells, their clinical applications are hindered by many research barriers. One of the major challenges is to obtain adequate number of cells as these cells were found to lose their potency during sub-culturing and at higher passages. One of the reasons behind the senescence and aging of MSCs during *in vitro* expansion is the decrease in telomerase activity [50]. It has been reported that human BM-MSCs become senescent during long-term culture, manifested by decline in differentiation potential, shortening of the telomere length and morphological alterations [51]. Similar results are also reported when MSCs derived from bone marrow and adipose tissues were progressively cultured at higher passages. The actual age of the cells in culture is usually determined by population doublings (PDs) time and MSCs colonies derived from a single cell has shown up to 50 PDs in 10 weeks [52], whereas others have reported 30 PDs in approximately 18 weeks [51]. However, culturing MSCs for a long time resulted in an increase in the probability of malignant transformation [53] and also showed decline in their multipotency. Early MSCs have proved higher differentiation ability to chondrocytes, adipocytes and osteocytes; however, at higher passages and on long-term culture, this differentiation property declines [54]. There are two vital compounds which influence MSCs’ properties during *in vitro* culturing, serum and growth factors, which are associated with malignant transformation of MSCs at higher passages [54]. In minimal media condition, MSCs culturing requires 10% heat-inactivated FCS, but in such culture conditions the MSCs retain some FCS proteins, which may evoke immunologic response *in vivo* [55]. Expanding MSCs in serum-free culture media showed a gradual decrease in differentiation potential and telomerase activity, but cells were resistant to spontaneous transformation and could be expanded at higher passages without any chromosomal alteration [54]. However, due to variation in culture media and growth factors used, the comparison of data is difficult.

### *In vitro* differentiation potential

hMSCs have the capacity to differentiate into all the three lineages, i.e. ectoderm, mesoderm and endoderm, with various potency by employing suitable media and growth supplements which initiate lineage differentiation (Table 2).

**Table 2:** *In vitro* differentiation potential of hMSCs

Source of hMSCs	*In vitro* differentiation potential	References
BM-MSCs	Osteocytes, chondrocytes, adipocytes	[13,40,43,47,60]
	Hepatocytes	[101]
	Cardiomyocytes	[84]
	Pancreatic cells	[106–108]
	Neuronal cells	[89,128]
AD-MSCs	Osteocytes, chondrocytes, adipocytes	[13,43]
	Hepatocytes	[172]
	Cardiomyocytes	[173]
	Pancreatic	[174]
	Neuronal cells	[90,91]
Dental tissues-derived MSCs	Osteocytes, chondrocytes, adipocytes	[18,46]
	Pancreatic cells	[109,112]
	Melanocytes	[19]
	Neuronal cells	[98,99]
UCB-MSCs	Osteocytes, chondrocytes, adipocytes	[13,28,43,56]
	Hepatocytes	[104,105]
	Pancreatic cells	[143]
	Neuronal cells	[97]
Limb bud-derived MSCs	Osteocytes, adipocytes	[21]
	Hepatocytes	[21]
	Neuronal cells	[21]
Wharton's jelly-derived MSCs	Osteocytes, chondrocytes, adipocytes	[30,31]
	Hepatocytes	[175]
	Neuronal cells	[88]
Skin- and foreskin-derived MSCs	Osteocytes, chondrocytes, adipocytes	[26,27]
	Myocytes	[26]

#### Mesodermal lineages

In addition to multipotency and expressions of cell surface markers, one of the determining properties of MSCs is to differentiate into mesodermal lineages. The *in vitro* differentiation into adipocytes, osteocytes and chondrocytes, confirmed by production of oil droplet, formation of mineralized matrices and expression of type II collagen respectively, has been evaluated by immunocytochemical, histochemical and PCR analysis [10,56–58]. Differentiation of MSCs into adipocytes is induced by proper media supplementations, which activate transcription factors (genes) responsible for adipogenesis. For adipogenesis, MSCs were cultured in growth medium supplemented with dexamethasone, indomethacine, insulin and isobutyl methyl xanthine for 3 weeks and the cells were analysed by accumulation of lipid droplets and expression of adipocytes-specific genes peroxisome proliferator-activated receptor γ (PPARγ), adipocyte protein 2 (ap2) and lipoprotein lipase (LPL) genes [10,59]. Induction of adipogenesis is characterized by two phases: determination phase and terminal differentiation phase [60]. During determination phase, the cells committed towards pre-adipocytes show similar morphology to fibroblasts and cannot be distinguished from their MSCs precursors; however, at terminal phase the pre-adipocytes become mature adipocytes and formed lipid droplets and express adipocytes-specific proteins [59]. Overall, adipogenesis is an ordered process, involving multiple signalling cascades which are further discussed later in the present review.

The classical method to differentiate MSCs into osteocytes is by culturing the cells with ascorbic acid, β-glyceralphosphate and dexamethasone for 3 weeks in growth conditioned media. The osteogenic induction of MSCs initiated mineral aggregation and showed increase in alkaline phosphatase activity at final week of differentiation [10]. These mineralized nodules were found positive for Alizarin Red and von Kossa staining. The process of osteogenesis starts with assurance of osteoprogenitor which first differentiate into pre-osteocytes and then finally differentiate into mature osteoblasts [61]. One of the most important indicating factors for osteogenesis is the expression of runt-related transcription factor 2 (Runx2) [61]; however, other transcription factors like osteonectin, bone morphogenic protein 2 (BMP2) and extracellular signal molecules along with Runx2 expression, are involved in this process. In the whole process of bone formation, first osteoblasts synthesize the bone matrix and then help in bone remodelling and mineral deposition.

The differentiation of MSCs into mesenchymal lineage is known to be controlled by diverse transcription factors and signalling cascades. Many investigators have reported that a correlation exists between adipogenesis and osteogenesis [62,63]. It was reported that a converse relationship exists between adipogenesis and osteogenesis during culturing with different media supplements. [64]. Several signalling pathways such as Hedgehog [65,66], NEL-like protein 1 (NELL-1) [63] and β catenin-dependent Wnt [67,68] are well manifested for pro-osteogenic and anti-adipogenic stimulations in MSCs, although there are various signalling cascades which demonstrate positive regulation of both adipo- and osteogenesis. Among them, one of the most familiar clinically-relevant molecule is BMP, which promotes MSCs differentiation and its osteogenic commitment [69,70] and also induce pro-adipogenic effects [71]. PPARγ and Runx2 are the key transcription factors which control the adipogenic and osteogenic signalling cascades and the expression of one transcription factor counteracts expression of other transcription factor [14,72].

Like the adipogenesis and osteogenesis, hMSCs have the potential to differentiate into mature chondrocytes. The first standard protocol for chondrocytes differentiation was established for MSCs derived from human bone marrow [73]. According to the standard protocol for chondrogenesis, cells were cultured in DMEM media supplemented with insulin transferrin selenium, linoleic acid, selenious acid, pyruvate, ascorbate 2-phosphate, dexamethasone and transforming growth factor-β III (TGF-βIII). The pre-induction stage of chondrogenic differentiation of MSCs resulted in the formation of pre-chondrocytes and expresses type I and type II collagens [74]. The expression of these genes and other adhesion molecules depends on the presence of soluble factors, i.e. TGF-β family (TGF-β1, TGF-β2 and TGF-β3) [75]. In the final step, pre-chondrocytes differentiate into mature chondrocytes and express chondrogenic transcription factors like Sox9, L-Sox5 and Sox6 [76,77]. In association with TGF-β1, other growth factors such as, insulin like growth factor-I (IGF-I) and BMP-2 were known to induce the differentiation of MSCs into chondrocytes [78]. In hMSCs, TGF-β1 interacts with Wnt/β-catenin pathways inhibits osteoblast differentiation and induce chondrogenesis [79]. When human AD-MSCs were treated with BMP-2, they differentiated into chondrocytes and expressed mature cartilage markers (type II collagen/GAG) [80]. Besides these growth factors, other hormones such as parathyroid hormone-related peptide (PTHrp) [81,82] and triiodothyronine (T3) also influenced chondrogenesis.

Like cardiomyocytes, MSCs can differentiate into other mesodermal lineages. Twenty years ago, the rat BM-MSCs were cultured with 5-azacytidine which resulted in the differentiation of these cells into multinucleated myotubes [83]. Later Xu et al. [84] treated human BM-MSCs with the same chemical and demonstrated that the cells differentiate into myocytes and were expressing myocyte-related genes, β-myocin heavy chain, α-cardiac actin and desmin with additional calcium–potassium-induced calcium fluxes. Human BM-MSCs also differentiate into skeletal muscles and smooth muscles when transfected with notch intracellular domain (NICD) [85] followed by treatment with TGF-β [86]. Yet the exact *in vivo* signalling mechanism which initiates the differentiation of hMSCs into myocytes is not completely understood and under investigation.

#### Ectodermal lineages

Despite the mesodermal origin, hMSCs have displayed the capacity of trans-differentiation into ectodermal lineages. The hMSCs isolated from different sources have demonstrated trans-differentiation into neuronal cells upon exposure to neural induction media supplemented with cocktails of growth factors. Several growth factors like hepatocyte growth factor (HGF), FGF and EGF were used in neuronal induction media cocktail and successfully obtained neuronal specific phenotypes, i.e. oligodendrocytes, cholinergic and dopaminergic neurons [87–91]. Barzilay et al. [89] reported that a transcription factor neurogenin-1 was found effective in the trans-differentiation of MSCs into neuronal protein expressing cells. In another study, a LIM homoeobox transcription factor 1 α (LMX1a) expression into human BM-MSCs resulted in differentiation to dopaminergic neurons [89]. When BM-MSCs were cultured in serum-free media with forskolin and cAMP, cells attained neuronal morphology and elevated the expression of neuronal-specific markers [92]. β-Mercaptoethanol (BME)- and nerve growth factor (NGF)-treated MSCs also differentiated into cholinergic neuronal cells [87]. Many studies have shown that factors like insulin, retinoic acid, bFGF, EGF, valproic acid, BME and hydrocortisone support neuronal differentiation of AD-MSCs [93,94]. Glial cell line-derived neurotrophic growth factors (GNDF), brain-derived neurotrophic factors (BDNF), retinoic acid, 5-azacytidine, isobutylmethylxanthine (IBMX) and indomethacin enhanced the MSCs differentiation into mature neuronal cells [95]. Gangliosides are glycosphingolipids which interact with EGF receptor (EGFR) and enhance osteoblast formation. However, reduction in gangliosides biosynthesis leads to inhibition of neuronal differentiation [96]. Human umbilical cord blood-derived MSCs (UCB-MSCs) co-transfected with telomerase reverse transcriptase (TERT) and BDNF revealed a longer life span and maintained neuronal differentiation which was effective in recovery of hypoxic ischaemic brain damage (HIBD) [97]. The dental derived MSCs, which originate from neural crest, successfully differentiated into mature neuronal cells [98,99]. hMSCs originate from mesoderm but have the potential to transdifferentiate into neural cells which can revolutionize the regenerative cell therapy in treating many neurological disorders.

#### Endodermal lineages

It was believed that hepatocytes could only be derived from the cells originating from endoderm and their progenitor cells. However, MSCs have revealed the capacity of trans-differentiation into hepatocytes and pancreocytes upon induction with their corresponding conditioned media. Human BM-MSCs were trans-differentiated into hepatocyte by using two steps protocol: differentiation step followed by maturation step. In differentiation step, cells were cultured in Iscove's modified Dulbecco's medium (IMDM) supplemented with EGF, bFGF and nicotinamide for a week. Finally during maturation step, differentiated human BM-MSCs were cultured with IMDM supplemented oncostatin M, dexamethasone and ITS^+^ (insulin, transferrin, selenium) premix which resulted in mature hepatocytes [100,101]. The hepatocyte-differentiated cells expressed liver-specific transcription markers, i.e. albumin, α-fetoprotein, nuclear factor 4 α (HNF-4α); however, the differentiation capacity remains inadequate for clinical application. Among these transcription factors, HNF-4α is an essential transcription factor for the morphological and functional differentiation towards hepatocytes [102,103]. When human UCB-MSCs were transduced with HNF-4α, it enhanced the differentiation capacity of the cells and increased expression of liver-specific markers [104]. In other studies, it was shown that valproic acid, which is histone deacetylase inhibitor, up-regulate the expression of hepatic marker through activation of protein kinase B (AKT) and extracellular signal-regulated kinases (ERK) [105].

Human BM-MSCs have been successfully differentiated into insulin producing β-cells *in vitro* and transplanted to streptozotocin-induced diabetic mice which corrected the hyperglycaemic condition [106,107]. The paracrine factors increase the differentiation and maturation of human BM-MSCs into pancreatic lineage without any genetic manipulation [108]. Human dental pulp stem cells also differentiated into insulin producing cells by induction with growth factors, i.e. acitvin A, sodium butyrate, taurine and nicotinamide [109]. Till now hMSCs derived from adipose, dental, umbilical cord, amnion, Wharton jelly and placental tissues have successfully differentiated into insulin producing β-cells [110–112]. These studies have revealed that hMSCs can differentiate into endodermal lineages which can transform the current traditional drug therapies to a future promising cell based therapies.

### Immunomodulatory features

Regarding clinical research on cellular therapy, it is very important to know about the immunomodulatory capabilities of MSCs. In the current era of cell therapy and transplantation, the infusion of MSCs and host compatibility is the main subject of interest. Due to low expression of MHC I and lack expression of MHC class II along with co-stimulatory molecules, like CD80, CD40 and CD86, MSCs are unable to bring substantial alloreactivity and these features protects MSCs from natural killer (NK) cells lysis [113]. The MSCs therapy might alleviate disease response by increasing the conversion from Th2 (T helper cells) response to Th1 cellular immune response through modulation of interleukin (IL)-4 and interferon (IFN)-γ levels in effector T-cells [114]. MSCs have the ability to inhibit the NK cells and cytotoxic T-cells by means of different pathways. The secretion of human leucocytes antigen G5 was also found helpful in the suppression of T lymphocytes and NK cells [115]. By the secretion of suppressors of T-cells development [116], inhibitory factors i.e. leukaemia inhibitory factor (LIF) [117] and IFN-γ [118] enhance immunomodulatory properties of MSCs. Moreover, it is observed that human BM-MSCs were not recognized by NK cells, as they expressed HLA-DR molecules [119]. When allogenic hMSCs were transplanted into patients, there was no production of anti-allogeneic antibody nor T-cell priming [120], but the cytotoxic immune factors were found to be involved in the lysis of MSCs [114,121]. In this situation, the IFN-γ act as antagonist of NK cells, i.e. IL-2-treated NKs are recognized to destroy MSCs whereas IFN-γ helps the MSCs to keep it safe from NKs [122]. In the same report, Jewett et al. [122] mentioned that along with the protection of MSCs from cytotoxic factors, IFN-γ also enhances the differentiation of these cells by nuclear factor kappa β (NFκB)-dependent and -independent pathway. Toll-like receptors (TLRs) are the key components of innate immune system, which is critically involved in the initiation of adaptive immune system responses. MSCs have the expression of TLRs that elevate their cytokines secretions as well as proliferation [123]. MHC class I chain-like gene A (MICA) together with TLR3 ligand and other immunoregulatory proteins kept the MSCs safe from NKs invasion [123]. Together with other properties, these immunomodulatory features makes MSCs one of the feasible stem-cells source for performing cell transplantation experiments.

### Human mesenchymal stem cells and chronic diseases

Considering the homing ability, multilineage potential, secretion of anti-inflammatory molecules and immunoregulatory effects, MSCs are considered as promising cell source for treatment of autoimmune, inflammatory and degenerative diseases. Efforts have been made to discuss the role of MSCs in treating chronic diseases in animal disease model (Table 3).

**Table 3:** hMSCs and chronic diseases

Disease	Clinical condition	Cell type	Species	Observations/Results obtained	References
Neurodegenerative diseases	ALS	AD-MSCs	Rat	Enhance pathological phenotype and enhance neuromuscular connections	[125]
	PD	BM-MSCs	Rat	MSCs found in hippocampus, cerebral and cortex of brain, increase level of tyrosine hydroxylase and DA	[88,129]
	AD	AD-MSCs	Mice	Increase Aβ-degradation enzyme secretion and expression of pro-inflammatory cytokines	[131]
		UCM-MSCs	Mice	Activate Tregs and increase neuronal survival	[134]
		BM-MSCs	Mice	Clear amyloid plaque, increase neuronal survival and enhanced cell autophagy pathway	[136]
Autoimmune diseases	RA	AD-MSCs	Mice	Elevation of the inflammatory response	[137]
		AD-MSCs	Mice	Th1/Th7 antigen-specific cells expansion, reduction in inflammatory chemokine and cytokines, increase secretion of IL-10	[138]
		BM-MSCs	Mice	Reduction in inflammatory chemokine and cytokines	[139]
	Type 1 diabetes	BM-MSCs	Mice	Formation of glucose competent pancreatic cells	[108]
		UM-MSCs	Mice	Differentiated into β-cells, produce human C-peptide in response to glucose challenge	[143]
Cardiovascular diseases	Myocardial infarction	BM-MSCs	Mice	Partially recompensed infarcted myocardium	[148,149]
	Acute myocardial infarction	UCB-MSCs	Mice	proliferating early and then differentiate into endothelial lineage	[153,154]

#### Neurodegenerative diseases

##### Amylotrophic lateral sclerosis

We previously discussed that MSCs have the ability to differentiate into neurons [87–99]. The first MSCs transplantation for neurodegenerative disorder was conducted in acid sphingomyelinase mouse model. After the injection of MSCs, there was a decrease in disease abnormalities and improvement in the overall survivability of the mouse [124]. Based on this experiment, a new study was designed to ascertain the potency of MSC transplantation into amylotrophic lateral sclerosis (ALS), a neurodegenerative disease that particularly degenerate the motor neurons and disturb muscle functionality [124]. The MSCs were isolated from the bone marrow of patients and then injected into the spinal cord of the same patients, followed by tracking of MSCs using MRI at 3 and 6 months. As a result, neither structural changes in the spinal cord nor abnormal cells proliferation was observed. However, the patients were suffering from mild adverse effects, i.e. intercostal pain irradiation and leg sensory dysesthesia which were reversed in few weeks duration. In another study, the AD-MSCs were genetically modified to express GDNF and then transplanted in rat model of ALS which improved the pathological phenotype and increased the number of neuromuscular connections [125].

##### Parkinson's disease

Parkinson's disease (PD) is a neurodegenerative disorder, characterized by substantial loss of dopaminergic neurons. The MSCs enhanced tyrosine hydroxylase level after transplantation in PD mice model [126]. MSCs by secretion of trophic factors like vascular endothelial growth factor (VEGF), FGF-2, EGF, neurotrophin-3 (NT3), HGF and BDNF contribute to neuroprotection without differentiating into neurocytes [127,128]. Now new strategies are being adopted like genetic modifications of hMSCs, which induce the secretions of specific factors or increase the dopamine (DA) cell differentiation. BM-MSCs were transduced with lentivirus carrying LMX1a gene and the resulted cells were similar to mesodiencephalic neurons with high DA cell differentiation [89]. Research group from the university hospital of Tubingen in Germany first time delivered MSCs through nose to treat neurodegenerative patients. The experiments were performed on Parkinson diseased rat with nasal administration of BM-MSCs [129]. After 4.5 months of administration, MSCs were found in different brain regions like hippocampus, cerebral, brain stem, olfactory lobe and cortex, suggesting that MSCs could survive and proliferate *in vivo* successfully [129]. Additionally, it was observed that this type of administration increased the level of tyrosine hydroxylase and decreased the toxin 6-hydroxydopamine in the lesions of ipsilateral striatum and substantia nigra. This novel delivery method of MSCs administration could change the face of MSCs transplantation in future.

##### Alzheimer disease

Alzheimer disease (AD) is one of the most common neurodegenerative disease. Its common symptoms are dementia, memory loss and intellectual disabilities. Till now no treatment has been established to stop or slow down the progression of AD [130]. Recently, researchers are in the search to reduce the neuropathological deficits by using stem cell therapy in AD animal model. It was demonstrated that human AD-MSCs modulate the inflammatory environment, particularly by activating the alternate microglia which increases the expression of Aβ-degradation enzymes and decreases the expression of pro-inflammatory cytokines [131]. Furthermore, it was observed that MSCs modulate the inflammatory environment of AD and inadequacy of regulatory T-cells (Tregs) [132] and later on it was reported that they could modulate microglia activation [133]. It was previously demonstrated that human UCB-MSCs activate Tregs which in turn regulated microglia activation and increased the neuronal survival in AD mice model [134]. Most recently, it was evidenced that MSCs enhanced the cell autophagy pathway, causing to clear the amyloid plaque and increased the neuronal survivability both *in vitro* and *in vivo* [135].

#### Autoimmune diseases

MSCs are also used to assuage immune disorders because MSCs have the capacity of regulating immune responses [1]. After revealing the facts that human BM-MSCs could protect the haematopoietic precursor from inflammatory damage [136], other hMSCs can be used for the treatment of autoimmune diseases.

##### Rheumatoid arthritis

Rheumatoid arthritis (RA) is a joint inflammatory disease which is caused due to loss of immunological self-tolerance. In preclinical studies on animal models, MSCs were found helpful in the disease recovery and decreasing the disease progression. The injections of human AD-MSCs into DBA/1 mice model resulted in the elevation of inflammatory response in the animal [137]. They further demonstrated that following the injections of AD-MSCs, the Th1/Th17 antigen-specific cells expansion took place due to which the levels of inflammatory chemokines and cytokines reduced, whereas this treatment increased the secretion of IL-10 [138]. Along with its anti-inflammatory function, IL-10 is an important factor in the activation of Tregs that controls self-reactive T-cells and motivates peripheral tolerance *in vivo* [138]. Similar to this, human BM-MSCs demonstrated the same results in the collagen-induced arthritis model in DBA/1 mice [139]. These studies suggest that MSCs can improve the RA pathogenesis in DBA/1 mice model by activating Treg cells and suppressing the production of inflammatory cytokines. However, some contradictions were reported in adjuvant-induced and spontaneous arthritis model, showing that MSCs were only effective if administered at the onset of disease, which suggests that on exposing to inflammatory microenvironment MSCs lost their immunoregulatory properties [140].

##### Type 1 diabetes

Type 1 diabetes is an autoimmune disease caused by the destruction of β-cells due the production of auto antibody directed against these cells. As a result, the quantity of insulin production reduces to a level which is not sufficient to control the blood insulin. It has been demonstrated that MSCs can differentiate into insulin producing cells and have the capacity to regulate the immunomodulatory effects [118]. For the first time, nestin positive cells were isolated from rat pancreatic islets and differentiated into pancreatic endocrine cells [141]. Nestin positive cells were isolated from human pancreas and transplanted to diabetic nonobese diabetic/severe combined immunodeficiency (NOD-SCID) mice, which helped in the improvement of hyperglycaemic condition [142]. However, these studies were found controversial and it was suggested that besides pancreatic tissues, other tissues can be used as an alternative for MSCs isolation to treat type 1 diabetes. Human BM-MSCs were found effective in differentiating into glucose competent pancreatic endocrine cells *in vitro* as well as *in vivo* [108]. Studies on UCB-MSCs presented a fascinating option for the use of these cells for insulin producing cells. It was demonstrated that UCB-MSCs behave like human ESCs, following similar steps to form the differentiated β-cells [143]. The most recent findings of Unsal et al. [144] showed that MSCs when transplanted together with islets cells into streptozotocin treated diabetic rat model enhance the survival rate of engrafted islets and are found beneficial for treating non-insulin-dependent patients in type 1 diabetes.

#### Cardiovascular diseases

For myocardial repair, cardiac cells transplantation is a new strategy which is now applied in animal models. MSCs are considered as good source for cardiomyocytes differentiation. However, *in vivo* occurrence of cardiomyocytes differentiation is very rare and *in vitro* differentiation is found effective only from young cell sources [145,146]. MSCs trans-differentiated into cardiomyocytes with cocktail of growth factors [84] were used to treat myocardial infarction and heart failure secondary to left ventricular injury [147]. The systematic injection of BM-MSCs into diseased rodent models partially recompensed the infarcted myocardium [148,149]. Furthermore Katrisis et al. [150] transplanted autologous MSCs along with endothelial progenitor cells and evidenced the improvement in myocardial contractibility, but they did not decrypt the mechanism which brought out these changes. Although MSCs are effective in myocardial infarction and related problems, but still cell retentivity in the heart is rapidly decreased, after 4 h of cells injection only 10% and after 24 h it was found approximately 1% cell retention [151,152]. Following this study, Roura et al. [153] reported that UCB-MSCs retained for several weeks in acute myocardial infarction mice, proliferated early and then differentiated into endothelial lineage. Most recently, transplantation of UCB-MSCs into myocardial infarction animal model along with fibronectin-immobilized polycaprolactone nanofibres were found very effective [154]. All these studies collectively indicate the role of hMSCs in cellular therapy of cardiac infarction and currently there are approximately 70 registered trials investigating the effect of MSCs therapy for cardiac diseases (clinicaltrials.gov).

### Homing of MSCs

Homing is the term used when cells are delivered to the site of injury, which is still challenging for cell-based therapies. Most of the time local delivery and homing of cells are found beneficial due to interaction with the host tissues, accompanied by the secretion of trophic factors [114]. There are a number of factors, like cells age, culturing conditions, cell passage number and the delivery method, which influence the homing ability of MSCs to the injured site.

Higher passage number decreases the engraftment efficiency of MSCs and it has been shown that freshly isolated MSCs had greater homing efficiency than the cultured cells. Besides this, the source from which MSCs are being isolated also influences the homing capacity of MSCs. While culturing MSCs, it was shown that oxygen condition, availability of cytokines and growth factors supplements in the culture media triggers important factors which are helpful in the homing of MSCs. Matrix metallo-proteases (MMPs), the important proteases which are involve in the cell migration, also plays important role in the MSCs migration [155]. The higher cell numbers and hypoxic condition of the culturing environment influence the expression of these MMPs [156]. The inflammatory cytokines, i.e. IL-1β, TNF-α and TGF-1 β, enhance the migration of MSCs by up-regulating the level of MMPs [155]. The next important factor is delivery method via which the MSCs are administered to the desired tissue. Intravenous infusion was the most commonly administered route [157], because if MSCs were administered systemically it will trap in the capillaries sheet of various tissues, especially in lungs [158]. That is the reason why most of the time intra-arterial injections of MSCs has been advised, but the most convenient and feasible way of MSCs transplantation is local injection to the site of injury or near the site of injury which provides more number of cells and increases its functional capacity.

The exact mechanism via which MSCs migrate and home to the injured site is still unknown, although it is believed that certain chemokine and its receptors are involved in the migration and homing of MSCs to the tissue of interest. MSCs express many receptors and adhesion molecules which assist in its migration process. The chemokine receptor type 4 (CXCR4) and its binding protein stromal-derived factor 1-α (SDF-1α) play a vital role in this process [159]. In order to know the homing capacity and to monitor the therapeutic efficiency of MSCs, *in vivo* tracking by non-invasive method are pre-requisite. Some advance techniques, i.e. single photon emission CT (SPECT), bioluminescence imaging (BLI), positron emission tomography (PET) were being applied for tracking the MSCs.

As we discussed earlier that MSCs have higher trans-differentiation potential and exhibits immunomodulatory features, but their off target homing, especially lodging in the lungs, is a major obstacle. There is need for in-depth study of MSCs homing mechanism and finding appropriate tracking without any negative effect on the cells and host.

### Cryopreservation and banking

From all the previous studies, it is obvious that the use of hMSCs for clinical applications will increase in future. For clinical applications, a large number of MSCs in an ‘off the shelf’ format are required. For this purpose, a proper set up of *in vitro* MSCs expansion and subsequent cryopreservation and banking are necessary to be established. This will provide unique opportunities to bring forward the potential uses and widespread implementation of these cells in research and clinical applications. Keeping in mind its use in future clinical and therapeutic applications, there is a need to ensure the safety and efficacy of these cells while cryopreserving and banking. For the selection of optimal cryopreservation media, uniform change in temperature during freezing and thawing, employed freezing device and long-term storage in liquid nitrogen are the indispensable factors to consider.

First considerable factor is the optimal cryopreservation media in which cells can maintain their stem cells abilities for long time. In the cryopreservation media, the cells require the animal base reagent, like FBS, as a source of their nutrients, but previous studies have suggested that animal proteins are difficult to remove from the hMSCs and that these resident protein may enhance adverse reactions in the patients who receive these cells for treatment [35]. Therefore, a serum-free media is substantial for the cryopreservation of MSCs and researchers have successfully used the serum-free media for cryopreservation of MSCs [160,161]. Most recently, human albumin and neuropeptide were used instead of FBS and MSCs maintained their cell survival and proliferation potential in the culture conditions. Additionally, cryoprotective agents (CPAs) are required for the cryopreservation media to prevent any freezing damage to cells. A large number of CPAs are available [162] among which DMSO is the most common CPAs used in cryopreservation of MSCs. However, DMSO is toxic to both humans and animals which make it complicated in the use of MSCs freezing for clinical applications and it has been showed that DMSO has bad effects in both animals and humans [163]. On the infusion of MSCs frozen in DMSO, patients develop mild complications like nausea, vomiting, headache, hypertension, diarrhoea and hypotension [164] and also severe effects like cardiovascular and respiratory issues were reported [165]. Due to these toxic effects, it is necessary to remove (washing with isotonic solutions) or replace DMSO with an alternate CPA. There are several methods along with the introduction of automated cells washing for the removal of DMSO from the frozen thawed cells [166]. Most recently for tissue cryopreservation, a new method was introduced using the mixture of 0.05 M glucose, 0.05 M sucrose and 1.5 M ethylene glycol in phosphate buffer saline [167], shown successful isolation and characterization of MSCs after 3 months of cryopreservation of the tissue. Hence, this method is without any DMSO and animal serum, but it is not yet applied for MSCs cryopreservation. From these findings, it is clear that for clinical grade cells, there is a need of a cryopreservation protocol either with low concentration of DMSO or to replace DMSO with non-toxic alternative.

For cryopreservation of MSCs, the second important factor is the freezing temperature rate. Mostly slow freezing at the rate of 1°C/min is the optimum rate for MSCs preservation [168]. For this purpose, current controlled rate freezers (CRFs) are suitable for controlling temperature, maintaining the rate of temperature during cryopreservation. These CRFs can be programmed to find out the exact temperature which the sample is experiencing during freezing [169]. Despite of these benefits, these CRFs lack the uniformity of temperature to all vials during large-scale banking of MSCs [170], so for large-scale banking, the development of advance CRFs are mandatory. Recently more advanced CRF, which provides unidirectional flow of cryogen to each sample, were created by Praxair Inc. On large-scale MSCs banking, along with the safe and efficient cryopreservation, the regulatory guidelines are also important. Like in the U.S.A., Food and Drug Administration (FDA) is responsible whereas in Europe, European Medicines Agency is responsible in Europe for supervising MSCs based cell therapy products.

### MSCs in clinical trials

**Figure 1:**
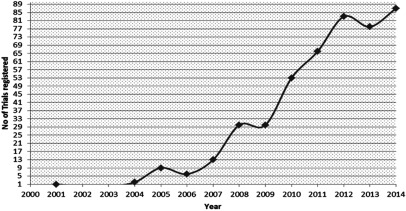
Number of clinical trials registered (per year) for MSCs based therapy (ClinicalTrials.gov)

**Figure 2:**
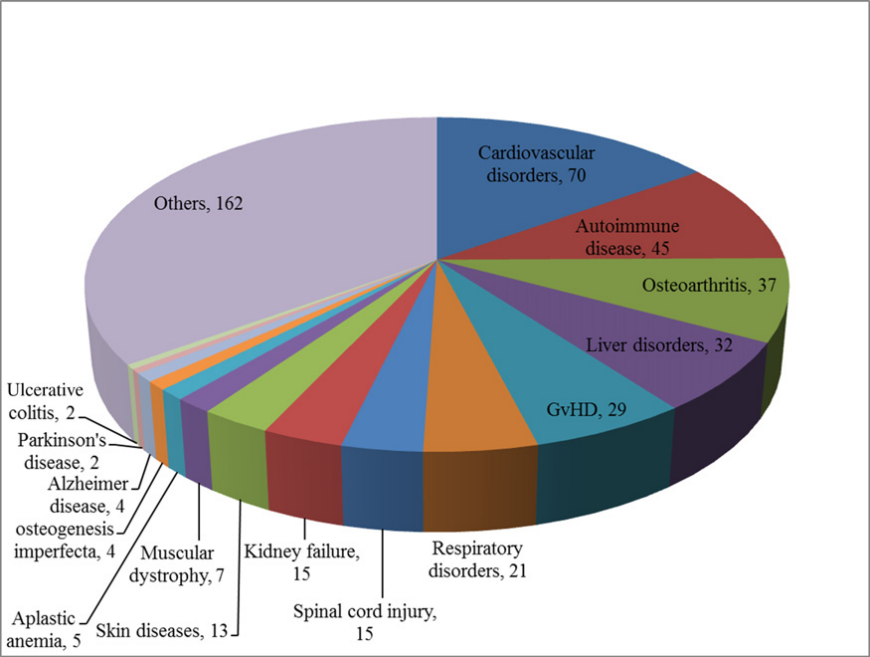
Number of common diseases registered for MSCs based cell therapy (ClinicalTrials.gov)

MSCs have a promising future in the world of clinical medicine and the number of clinical trials has been rising since the last decade. Along with preclinical studies, MSCs have been found to be persuasive in the treatment of many diseases [1]. A large number of clinical trials have been conducted and this trend is gradually increasing (Figure 1). Currently, there are 463 registered clinical trials in different clinical phases (phase I, II etc.), evaluating the potential of MSC-based cell therapy throughout the world (ClinicalTrials.gov). Most of these trials are phase I/II studies and combination of phase II/III studies, whereas very small numbers of these trials are in phase IV or phase III/IV. Among 463 registered trials, 264 trials are in open status which is open for recruitment whereas 199 trials are closed; out of which 106 studies are completed whereas the rest are in active phases. Clinical trials conducted with MSCs showed very less detrimental effects; however, few of them showed mild adverse effects. Due to immunomodulatory properties, MSCs have been used in many human autoimmune disease clinical trials. However, the exact mechanism by which MSCs regulate the immune response is unclear [171]. To date, 45 autoimmune-disease clinical trials have been registered, out of which seven are completed, 22 are open for recruitment whereas the rest are in active phases (ClinicalTrials.gov). Similarly 70 trials are registered for cardiovascular diseases, 37 for osteoarthritis, 32 for liver disorders, 29 for graft versus host disease (GvHD), 21 for respiratory disorders, 15 for spinal cord injury, 15 for kidney failure, 13 for skin diseases, seven for muscular dystrophy, five for aplastic anaemia, four for Osteogenesis imperfecta, four for AD, two for PD, two for ulcerative colitis and rest are for other diseases (Figure 2). Although the progress of clinical studies so far registered is slow (only seven studies with final results), but the efficient use of MSCs in large clinical trials with upcoming promising results have proven MSCs as boon for regenerative medicine.

## FUTURE PROSPECTS

Recent breakthrough discoveries in engineering MSCs have made it an ideal source for future cell therapy in regenerative medicine. MSCs adaptability to the exposed environment has made them an impressive source for disease treatment, though the full understanding of MSCs mechanism is still in their preliminary stages. After performing a large number of preclinical trials, the human clinical trials of MSCs are now on its way to success and many trials have been successfully accomplished (clinicaltrials.gov). During the last decade, many experimental and clinical assays had been developed; however, a number of questions related to MSCs biology are unsolved. These are related to MSCs survival and homing capacity after transplantation, the relationship between the host immunity and MSCs, the route of administration (local or systemic) and whether the properties like proliferation, differentiation and trans-differentiation are maintained after *in vivo* transplantation. Several reports have documented the successful transplantation, differentiation and homing of hMSCs but their effect in the concerned disease is due to secretion of cytokines rather than direct effect of MSCs. Furthermore the mechanism underlying migration of MSCs remains to be clarified, although evidence suggests that both chemokines and their receptors and adhesion molecules are involved in this process [37]. The future MSCs research should focus on finding more suitable markers to isolate the source-specific MSCs, basic understanding of growth regulators in differentiation and trans-differentiation and site-specific homing that can revolutionize the cell regeneration therapy. Moreover, to reduce the risk of oncogenic transformation special attention should be paid to the genetic safety of cell preparation. Nevertheless, an active research should focus on bio-banking in a large scale to use them in the future by developing a novel CPA/protocol without hampering their basic characteristics.

## CONCLUSION

hMSCs are not only easy to isolate but they also retain their ability to expand for long period of time without losing its characteristics. However, apart from mesodermal lineages, they have the capacity to trans-differentiate into ectodermal and endodermal lineages. Moreover, hMSCs have the immunomodulatory properties as they secrete certain cytokines and immune relevant receptors to modify the host immune environment. All these properties of MSCs make them distinct from other stem cells and can be used in future cell replacement therapy. Many preclinical and clinical studies were performed using hMSCs in treatment of chronic diseases like neurodegenerative diseases, autoimmune and cardiovascular diseases, but still there are questions that have to be answered before using hMSCs on large clinical scale. Firstly, the safety issues of MSCs should be solved, because after MSCs administration, mild adverse effects were observed and the most severe is that unfortunately long-term cultured MSCs promote tumour growth and metastasis. Secondly, quality control: before directly applying MSCs for *in vivo* transplantation, additional tests are needed to perform, like cell viability, endotoxin assays and oncogenic tests. Depending upon the severity of disease, an optimal dose and specific administration time is needed to be decided. The third and most important is clinical grade production of MSCs, because for clinical use of MSCs a large number of cells are required, for which *in vitro* expansion is vital, but MSCs at higher passages could lead to cell transformation. To conclude, though adult-derived hMSCs are a favourite choice, but before hMSCs can be used on large-scale clinical applications for cell therapy, there is a need for completely understanding the underlying mechanisms that regulate and modulate these MSCs.
